# Temporal prediction errors modulate task-switching performance

**DOI:** 10.3389/fpsyg.2015.01185

**Published:** 2015-08-25

**Authors:** Roberto Limongi, Angélica M. Silva, Begoña Góngora-Costa

**Affiliations:** ^1^UDP-INECO Foundation Core on Neuroscience, Diego Portales University, Santiago, Chile; ^2^Instituto Venezolano de Investigaciones Lingüísticas y Literarias “Andres Bello”, Caracas, Venezuela; ^3^Escuela Lingüística de Valparaíso de la Pontificia Universidad Católica de Valparaíso, Chile; ^4^Escuela de Fonoaudiología, Facultad de Medicina, Universidad de Valparaíso, Chile

**Keywords:** prediction errors, predictive coding, response inhibition, insular cortex, cognitive neuroscience

## Abstract

We have previously shown that temporal prediction errors (PEs, the differences between the expected and the actual stimulus’ onset times) modulate the effective connectivity between the anterior cingulate cortex and the right anterior insular cortex (rAI), causing the activity of the rAI to decrease. The activity of the rAI is associated with efficient performance under uncertainty (e.g., changing a prepared behavior when a change demand is not expected), which leads to hypothesize that temporal PEs might disrupt behavior-change performance under uncertainty. This hypothesis has not been tested at a behavioral level. In this work, we evaluated this hypothesis within the context of task switching and concurrent temporal predictions. Our participants performed temporal predictions while observing one moving ball striking a stationary ball which bounced off with a variable temporal gap. Simultaneously, they performed a simple color comparison task. In some trials, a change signal made the participants change their behaviors. Performance accuracy decreased as a function of both the temporal PE and the delay. Explaining these results without appealing to *ad hoc* concepts such as “executive control” is a challenge for cognitive neuroscience. We provide a predictive coding explanation. We hypothesize that exteroceptive and proprioceptive minimization of PEs would converge in a fronto-basal ganglia network which would include the rAI. Both temporal gaps (or uncertainty) and temporal PEs would drive and modulate this network respectively. Whereas the temporal gaps would drive the activity of the rAI, the temporal PEs would modulate the endogenous excitatory connections of the fronto-striatal network. We conclude that in the context of perceptual uncertainty, the system is not able to minimize perceptual PE, causing the ongoing behavior to finalize and, in consequence, disrupting task switching.

## Introduction

Bayes-based theories of brain function state that the brain is a predictive machine and that perception is no more than a prediction of the sensorium’s causes (Friston and Stephan, 2007; Friston, 2009, 2010; Friston and Kiebel, 2009; Daunizeau et al., 2012). Predictions produce prediction errors (PEs, the differences between the predicted and the actual events). In general, PEs are considered to drive both inference and learning. In predictive coding, this is equivalent to regarding the brain as a hierarchical Bayesian filter (Friston and Stephan, 2007; Friston, 2009, 2010; Friston and Kiebel, 2009; Daunizeau et al., 2012) whereas in associative learning the classic Rescorla–Wagner model (Rescorla and Wagner, 1972) calls upon reward PEs to learn the value of stimuli or actions. However, Bayesian models of associative learning also include PEs as a driving variable (Kruschke, 2008; Gershman et al., 2010). More cognitive models also state that PEs drive higher order cognition such as uncertainty-related cognitive control and learning (Carter et al., 1998; O’Reilly et al., 1999; Cohen et al., 2002; Brown and Braver, 2005; Alexander and Brown, 2010, 2011). Moreover, PEs might play a central role in explaining psychotic disorders (Braver et al., 1999; Adams et al., 2012; Bastos-Leite et al., 2015) and intersubject variability in social cognition of patients with brain damage (Limongi et al., 2014).

**FIGURE 1:**
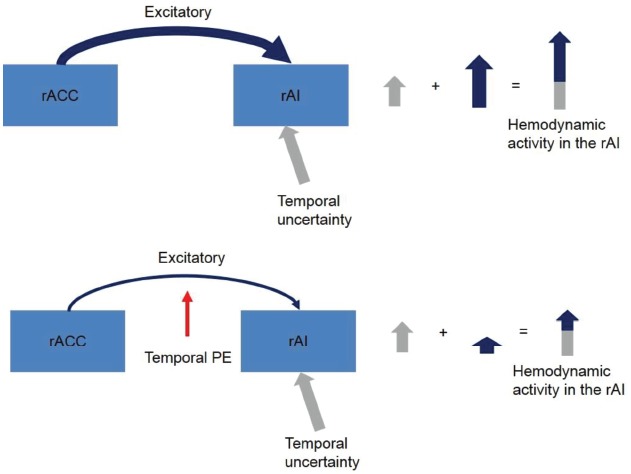
**Driving and modulatory effects of temporal uncertainty and temporal PEs in the rACC-rAI coupling as reported by Limongi et al. (2013).** When participants accurately predict an event’s onset time, the activity of the rAI increases. Additional insular activation is provided by afferent excitatory projections from the rACC. This extra excitatory effect is dampened by temporal PEs which suggests that when participants fail to accurately predict an event’s onset time the performance of an unexpected secondary-task demand decreases. Notice that in DCM endogenous connections are assumed excitatory.

In our recent neurophysiological study (Limongi et al., 2013), we identified a conjoint effect of uncertainty and PEs as driving and modulatory inputs of brain regions. In dynamic causal models of imaging data, a driving input is modeled as an experimental effect that directly drives the activity of a region whereas a modulatory effect is modeled as a change in the connection strength between two regions (Kahan and Foltynie, 2013). In our previous study, the participants performed temporal predictions with different levels of temporal uncertainty, and we found that temporal uncertainty drove the activity of the right anterior insular cortex (rAI) when the participants predicted the onset time of an event. However, we also found that the temporal PEs negatively modulated the excitatory (as assumed in dynamic causal models) connection strength between neurons of the right anterior cingulate cortex (rACC) and neurons of the rAI. This negative modulatory effect counteracted the driving effect of temporal uncertainty (Figure 1).

When we are certain about an event’s onset time, we anticipatorily prepare the behavior that we will execute at the event’s onset, we engage *temporal preparation* (Nobre et al., 2007; Fischer et al., 2012; Los, 2013; Rohenkohl et al., 2014). However, if after preparing a behavior we suddenly need to change the planned action with another, this time, “unprepared” behavior (e.g., stepping back from a road crossing when a walk signed changes unexpectedly) we face uncertainty because we are not expecting a task-switching demand. As mentioned, temporal PEs exert a negative modulatory effect on the excitatory cingulate-insular coupling which causes the activity in the rAI to decrease. Therefore, we should expect a PE-driven dampening effect on task-switching performance accuracy (e.g., changing a prepared behavior when a change demand is not expected) when we face perceptual uncertainty.

The above inference, however, is far from conclusive because, on one side, the activity of the rACC is also associated with behavioral contingencies explained in terms of conflict monitoring (Botvinick et al., 1999), cognitive control (Brown, 2008, 2011; Alexander and Brown, 2010), general attention (Carter et al., 1998), attention for learning (Bryden et al., 2011), and response inhibition (Aron and Poldrack, 2006). On the other side, the activity of the rAI has also been associated with behavioral contingencies explained in terms of attention (Eckert et al., 2009; Menon and Uddin, 2010; Nelson et al., 2010), response inhibition (Aron and Poldrack, 2006; Aron et al., 2014; Cai et al., 2014), and other forms of uncertainty (Preuschoff et al., 2008; Schultz et al., 2008; Bossaerts, 2010; Jones et al., 2010, 2011; Sarinopoulos et al., 2010; Payzan-LeNestour and Bossaerts, 2011, 2012; Payzan-LeNestour et al., 2013; Symmonds et al., 2013; Nursimulu and Bossaerts, 2014). In other words, the sole fact that the activity in the rAI is negatively modulated by temporal PEs is not sufficient to conclude that temporal PEs modulate an action update (i.e., a change in a prepared behavior). Otherwise, we would be committing a reverse inference fallacy (Poldrack, 2006, 2011). This neurophysiology-driven hypothesis needs specific behavioral test. In this work, we show that perceptual uncertainty compromises task switching or action selection when subjects have to inhibit a prepotent response and replace it with a new action. We will refer to this as *task switching* and examine the effect of perceptual uncertainty on task switching in terms of performance accuracy. In brief, subjects were required to report a perceptual decision at a particular peristimulus time. We introduced perceptual uncertainty by increasing the delay (i.e., temporal gap) between the perceptual decision and the time of response. Crucially, this was repeated with and without a task-switching demand during response preparation.

Our hypothesis is based upon predictive coding accounts of sensorimotor integration—and in particular active inference. We hypothesize that increasing perceptual uncertainty (by increasing the temporal gap) would compromise task switching and reduce performance accuracy. Based upon our previous neuroimaging findings, we suppose that this effect would be mediated by an encoding of uncertainty or precision. In brief, we argue that greater temporal gaps (and subsequent uncertainty) would have two consequences. First, there would be an increase in behavioral PEs in terms of the timing of the response. Second, this increased uncertainty or decreased precision would result in a decreased sensitivity of the rAI to ascending PEs. The subsequent reduction of precise predictions about action selection would reduce task switching and be revealed as a drop in response accuracy—when, and only when, task switching is necessary.

## Materials and Methods

### Participants

Sixteen right-handed students (five males, *M* age = 22.7 years) signed an informed consent form and participated in the study. The study was conducted fulfilling the ethical principles for medical research involving human subjects comprised in the Declaration of Helsinski and approved by the Ethics Committee of Instituto Pedagógico de Caracas.

### General Task Description

The participants had to report whether the color of two balls were the same or different when cued to respond a period of time after the decision was made. This delay or temporal gap was progressively increased to induce uncertainty about when the response would be cued. A trial comprised the appearance of two balls, where one ball moved toward a center ball from the periphery of the screen. After the first ball touched the second ball, the second ball bounced off with a variable temporal gap. Crucially, the balls could switch their colors shortly before touching. This meant that some trials required both the inhibition of the prepotent response to the initial colors and a preparation of a new response.

### Stimuli and Procedure

**FIGURE 2:**
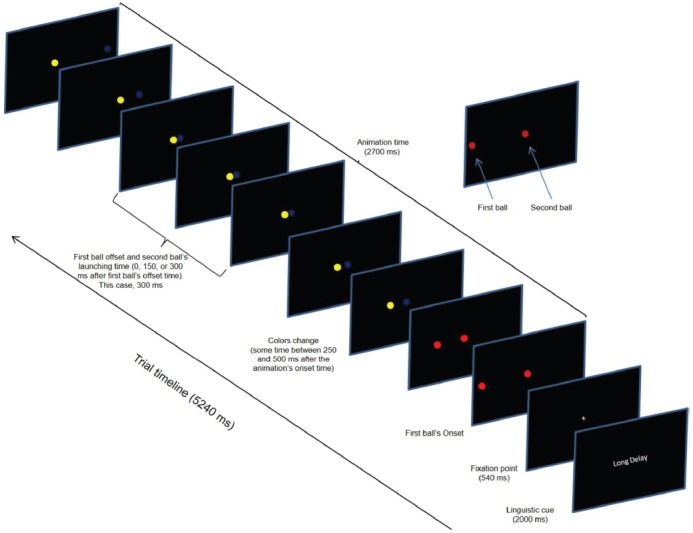
**Timeline of a single experimental trial depicting the ***long delay*** temporal gap during the ***change*** condition of the task**.

A single trial comprised three events: linguistic cue (2000 ms), fixation point (540 ms), and visual animation (2700 ms). The linguistic cue informed on the magnitude of the temporal gaps (“no delay,” 0 ms; “short delay,” 150 ms; and “long delay,” 300 ms). The fixation point announced the animation’s onset. At the animation’s onset, two colored balls (1.30° of visual angle in diameter) simultaneously appeared on the left and center of a computer screen. Then, the left-most ball (first ball in Figure 2) moved to the center of the screen at a constant speed (17.32 deg/s) until it stopped 900 ms later at the edge of the second ball. After a delay (temporal gap) of 0, 150, or 300 ms, the right-most ball (second ball in Figure 2) began moving to the right.

The participants had to press a response key when they predicted the second ball’s onset time. They pressed the “S” key if the balls’ colors were the same and the “D” key if the colors were different.

Critically, there were three task conditions: change, false-alarm, and no change. Our condition of interest was the change condition, but the false-alarm and the no-change conditions were included as control conditions and to prevent the participants from anticipating a task-switching demand which would improve their performance (Jahfari et al., 2012). Each task condition comprised 33% of the trials. In the change condition, the balls’ colors changed at some random time within a time window between 250 and 500 ms after the animation’s onset time. For example, if the initial colors were “red” and “red” they changed to “blue” and “white.” We will refer to the change time of the balls’ colors as the change-signal onset time (CSO). In the false-alarm condition, the balls changed in color, but the relational value remained the same. For example, if the initial colors were “red” and “white” (for the first and second ball respectively) they could change to “yellow” and “blue.” Notice that despite this change, the colors’ relational value (i.e., different) was the same. In the no-change condition, the balls’ colors did not change. Four colors were used (red, white, blue, and yellow). The stimulus delivery program randomly chose the combination of colors. The program also randomly varied the initial positions of the balls in the horizontal axis across trials; however, the initial distance between the balls remained constant across trials. Figure 2 shows the sequence of events in a single trial.

The experimenter explicitly instructed the participants to press the appropriate key just at the “exact” onset time of the second ball. Eight participants used the index finger to press the “S” key (middle finger to press the “D” key) whereas eight participants used the middle finger to press the “S” key (index finger to press the “D” key). The participants used the same hand in all of the trials. The dependent variables of interest were the response accuracy based upon the balls’ relational value and the absolute temporal PEs ( |response time – second ball’s onset time| ). Regardless of the duration of the temporal gap, the subjects sometimes made predictions before the second ball’s onset time (early predictions) and sometimes after the second ball’s onset time (late predictions). Young et al. (2005) showed that the absolute value of the temporal PE would better account for the effect of the temporal gaps than the relative (early/late) values. Moreover, we recently found that the absolute value of the temporal PEs better accounts for the neurophysiological effects of temporal gaps estimation than the relative values (Limongi et al., 2013). Notice that the absolute value of the behavioral PEs is related to their squared values. This means that the absolute values can be taken as a proxy for the precision (inverse variance) of behavioral response times.

### Design

We constructed a 3 × 3 factorial design: temporal gaps (with three levels: no delay, short delay, and long delay) times tasks (with three levels: change, no change, and false alarms). Each participant performed 450 trials (50 trials/condition) divided into 10 blocks (45 trials/block and five randomly intermixed trials per condition within each block). The participants also performed a familiarization block. Between blocks, a display message encouraged the participants to relax during a short break and to decide when to continue with the experiment. The experimenter provided feedback to the participants only during the familiarization block. The stimulus delivery program was E-Prime 2.0 (Psychology Software Tools, Pittsburgh, PA, USA).

### Data Analysis

To verify that the temporal gaps actually produced different PEs beyond random fluctuation, we performed a simple linear-mixed effects regression analysis with all of the valid responses. To make sure that we considered *predictions* of the second ball’s onset time rather than *reactions* to the second ball’s motion, we excluded late predictions if these were greater than 200 ms. This exclusion criterion yielded 91% of valid responses. We regressed the absolute PEs against the temporal gaps and included the subjects as a random effect.

To specifically test our hypothesis, we fit a series of mixed-effects linear models to the task-switching performance accuracy. More specifically, we defined a model space with four models representing our hypothesis and one additional model representing an alternative hypothesis. All of the models included subjects as a random effect.

First, it is possible that task-switching performance accuracy is disrupted by PEs but not by temporal gaps. Model 1a represented this possibility. It comprised the main effect of task, the main effect of PE, and the Task × PEs interaction. PEs were indexed in term of Vincentiles (Balota and Yap, 2011). At a subject level, the distribution of PEs was ordered and divided into 10 Vincentiles. Large Vincentiles represented large PEs.

Second, it is possible that the accuracy in sudden task switching is affected not only by the PEs, but also by temporal gap-induced uncertainty. Model 2a included all of the effects of model 1, the main effect of temporal gap, and the Temporal Gap × Task interaction.

Third, although the temporal window comprising the CSO was constant across temporal gaps, the CSO varied with respect to the second ball’s onset time. Therefore, it is possible that the CSO also accounts for some proportion of variance (Verbruggen et al., 2008). To model this possible confounding variable, we constructed two additional models (models 1b and 2b) by adding the Task × CSO interaction to the effects of models 1a and 2a.

Fourth, it is possible that neither PE nor temporal gap account for the decrease in task-switching performance accuracy. Alternatively, it is possible that only the CSO accounts for this effect. Therefore, model 3 included the main effect of task, the main effect of CSO, and the Task × CSO interaction.

To select the best model, we relied upon the models’ corrected Akaike information criterion number (AIC_c_) as a measure of the best compromise between generalizability, complexity, and goodness of fit (Myung, 2000; Pitt et al., 2002; Myung and Pitt, 2004; Myung et al., 2009). We also included the relative merits of the different models in terms of their Akaike weights (Wagenmakers and Farrell, 2004; Anderson, 2008). The Akaike weight (*w*) of a model *i* is defined by

(1) $$w_{i}=\frac{e^{\frac{-1\Delta i}{2}}}{\Sigma_{r=1}^{r}e^{\frac{-1\Delta r}{2}}}$$

where, Δ_*i*_ = *AICc_i_* – *AICc_min._*

Notice that the Akaike weight of a specific model changes depending on the number of competing models (i.e., the model space). Moreover, we complemented the models comparison strategy with traditional *F* tests on the fixed effects.

## Results

**FIGURE 3:**
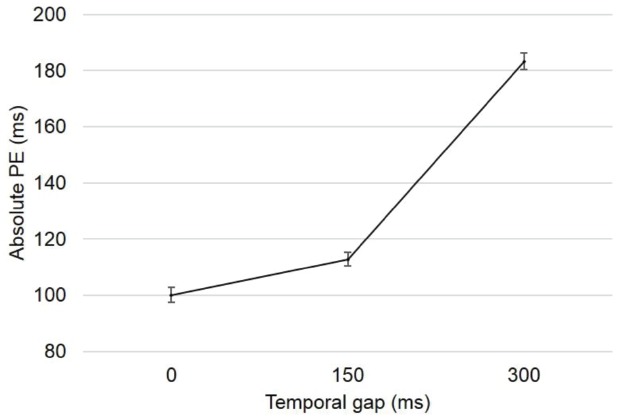
**Absolute PEs as a function of the temporal gaps**.

A simple linear mixed-effects regression model shows that, as expected, the absolute values of the PEs increased as a function of the temporal gap (β = 0.26, SE = 0.01, Figure 3), which replicates previous results (Young et al., 2005; Limongi et al., 2013). The slope (β) indicates that the absolute PE increased 0.26 ms per each millisecond of temporal gap increment.

**FIGURE 4:**
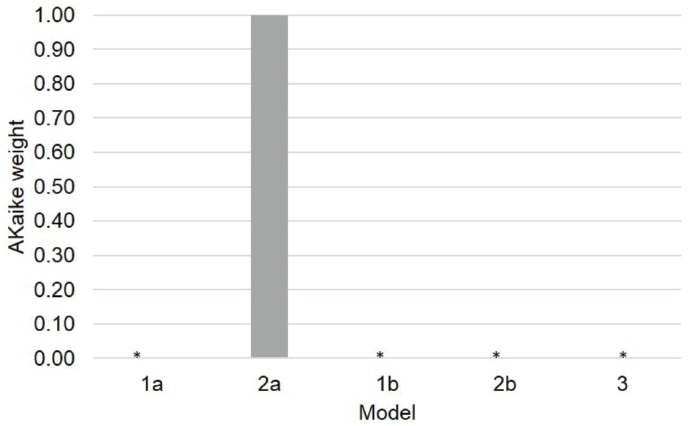
**Akaike weights of the hypothesized models.** The Akaike weights show the support that each model receives from the data relative to all of the other models within a specific model space.*Akaike weight approaches 0.

The models comparison procedure shows that model 2a (AIC_c_ = 6301) better accounts for the observed effects than the other three models (AIC_c_ model 1a = 6351, AIC_c_ model 1b = 6391, AIC_c_ model 2b = 6340, AIC_c_ model 3 = 6561). Figure 4 shows the Akaike weights of the models. Clearly, Model 2a surpasses all of the other models in the defined model space. Therefore, we selected model 2a as the simplest model that best fits the collected data and best generalizes to other data samples. The fixed effects tests confirmed the main effect of task, *F*(2, 6571) = 192.2, *p* < 0.0001; the main effect of temporal gap, *F*(1, 6572) = 39.6, *p* < 0.0001; the main effect of Vincentile, *F*(1, 6573) = 22.91, *p* < 0.0001, the Temporal Gap × Task interaction, *F*(2, 6571) = 36.03, *p* < 0.0001; and the Vincentile × Task interaction, *F*(2, 6571) = 27.28, *p* < 0.001.

**FIGURE 5:**
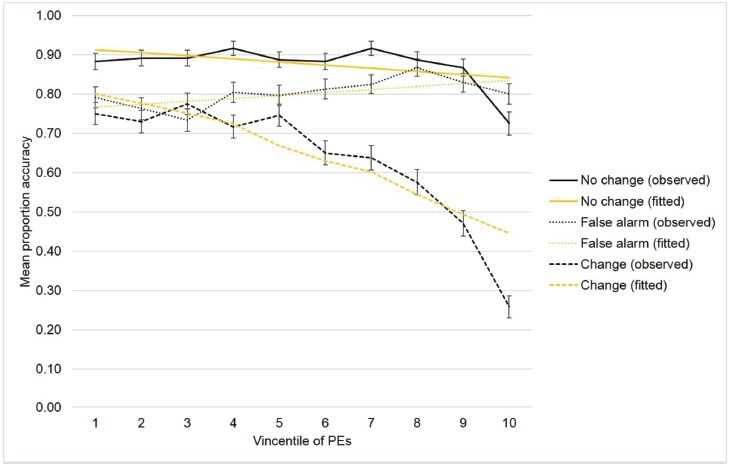
**Observed and fitted performance accuracy as a function of the PEs across the three task conditions.** PEs are represented in terms of Vincentiles.

Figure 5 shows the observed effects and the fitted lines as yielded by the parameters estimates (Table 1) of the winning model. The disrupting effect of the PEs on task-switching performance accuracy is fairly evident. Specifically, the slope of the regression line between Vincentile and color comparison accuracy was steeper for the change condition than for both the non-change and false-alarm conditions, meaning that the task-switching performance accuracy strongly decreased as a function of the temporal PEs. Finally, the parameter estimates also show that the slope of the regression line between temporal gap and the color comparison accuracy was steeper for the change condition than for both control conditions. It is relevant that the effects of PEs and temporal gaps were not collinear as verified by the small variance inflation factors (VIF).

**TABLE 1:** **Parameters estimates of the linear mixed-effects model**.

**Parameter**	**Estimate**	**SE**	**DF**	***t* Ratio**	**P**	**Lower 95%**	**Upper 95%**	**VIF**
Intercept	0.8618	0.0276	19	31.19	<0.0001	0.8040	0.9196	
Task [change]	–0.1221	0.0067	6571	–18.3	<0.0001	–0.1352	–0.1090	1.328
Task [false-alarm]	0.0197	0.0067	6571	2.92	0.004	0.0065	0.0328	1.327
Task [change] × (Vincentile-5.16315)	–0.0175	0.0026	6571	–6.71	<0.0001	–0.0226	–0.0124	1.534
Task [false-alarm] × (Vincentile-5.16315)	0.0158	0.0026	6571	6.06	<0.0001	0.0107	0.0210	1.506
Vincentile	–0.0088	0.0018	6572	–4.79	<0.0001	–0.0124	–0.0052	1.142
Temporal gap	–0.0003	0.0000	6571	–6.3	<0.0001	–0.0003	–0.0002	1.142
Task [change] × (temporal gap-144.473)	–0.0005	0.0001	6571	–8.45	<0.0001	–0.0006	–0.0004	1.547
Task [false-alarm] × (temporal gap-144.473)	0.0003	0.0001	6571	5.02	<0.0001	0.0002	0.0004	1.520

Vincentile and temporal gap values are mean centered.

## Discussion

In the causality literature, there is a well documented hypothesis stating that temporal contiguity of dynamics events is strongly associated with causal attribution and temporal prediction (Young et al., 2005; Young and Sutherland, 2009). Moreover, anticipatory (i.e., predictive) smooth pursuit eye movements are strongly associated with both temporal contiguity and causal attribution (Badler et al., 2010, 2012). This might suggest an alternative hypothesis on the observed increased in PEs associated with the increase in temporal gaps: Violation of causality rather than temporal uncertainty would induce temporal PEs. Although this alternative hypothesis deserves further studies, the independent contributions of both behavioral PEs and temporal gaps on task-switching performance accuracy are fairly supported by the data, providing behavioral evidence to the neurophysiologically motivated hypothesis that temporal PEs modulate unexpected task-switching performance.

A challenge to cognitive neuroscience is proposing brain-based mechanisms of cognition without appealing to *ad hoc* constructs such as a “central executive” or a “homunculus” (Hazy et al., 2006, 2007; O’Reilly and Frank, 2006). With this challenge in mind, we think that our results are entirely consistent with the predictive coding hypothesis: the experimental manipulation of delay (temporal gaps) induces a delay-specific encoding of uncertainty and precision. The ensuing reduction in precision explains the increase in behavioral PEs and reduces task-switching performance accuracy (through a decreased sensitivity to ascending PEs). In other words, in the absence of precise information, the brain relies on its prior beliefs and is more likely to emit prepotent responses. Crucially, the brain knows when sensory information is likely to be imprecise. This computational explanation fits comfortably with the decreased sensitivity of the rAI to ascending connections when stimuli have greater temporal uncertainty or less precision (because precision is thought to be encoded by the gain or postsynaptic sensitivity of neurons encoding PEs). Following, we expand upon this explanation. First, we will introduce general concepts of the predictive coding approach. Second, we will propose a neurophysiological model that would give rise to these behavioral results.

### Predictive Coding: Free Energy and the Hierarchical Minimization Process of PEs

The predictive coding theory of brain function defines perception as *exteroceptive* predictions (Adams et al., 2012). A percept is a hypothesis of the sensory data, and the perception process ends with the best hypothesis at hand in terms of Bayes optimal estimates of the sensorium’s causes. The mechanism through which the organism reaches this optimal hypothesis comprises the minimization of PEs as a hierarchical process.

**FIGURE 6:**
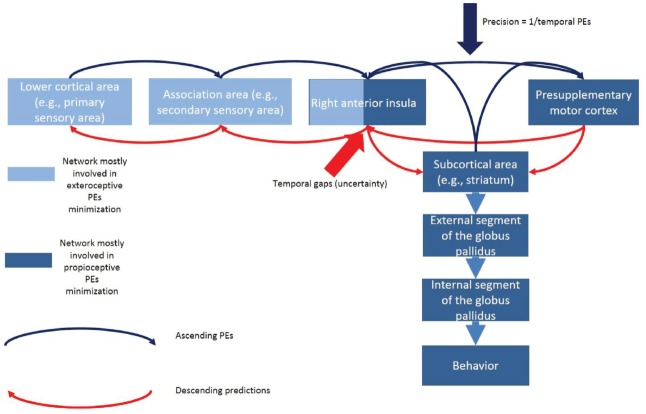
**Hypothetical model of the exteroceptive and proprioceptive PEs minimization in the context of task switching during temporal predictions.** The rAI links a circuit mostly engaged in minimization of exteroceptive PEs (associated with temporal predictions) with a circuit mostly engaged in the minimization of the proprioceptive PEs (associated with the control of action). Temporal uncertainty would drive the activity of the exteroceptive circuit whereas temporal PEs would modulate the effective connectivity of the fronto-basal ganglia network (the proprioceptive circuit). We hypothesize that whereas the activity of the rAI would facilitate temporal estimation, temporal PEs would negatively modulate the behavior-change performance. The net effect would be the execution of the anticipatorily prepared (but innacurate) behavior which would release proprioceptive-related free energy that could not be minimized via the exteroceptive circuit.

A hierarchical minimization process assumes that higher cortical areas (e.g., the prefrontal cortex) sends prediction signals to lower cortical areas and subcortical areas (e.g., primary visual cortex and fronto-basal ganglia circuits). At a given cortical level, the internal neural circuit (i.e., within the six-layer cannonical cortical column) computes a PE. This PE is sent forward to higher levels in the hierarchy (e.g., secondary visual area) to revise higher level representations. These updated representations then reciprocate descending or backward predictions to suppress PEs at the lower level. This process continues at all hierarchical levels until the PE has been minimized throughout the hierarchy (Figure 6).

The minimization of PEs gains physiological meaning in terms of free energy minimization. The free energy principle states that an organism tends to change its internal state to minimize free energy (Friston and Stephan, 2007). The free energy principle is congruent with the physiological tendency of an organism to reach equilibrium which is referred to as homeostasis. Therefore, free energy minimization is an adaptive “goal” of an organism while interacting with the environment. Critically, the minimization of the sensory PEs (i.e., perception) is only one mechanism available to this end.

Predictive coding also proposes that “action” or, in general, “behavior” is another way to minimize PEs (Friston et al., 2006, 2010), and, in consequence, free energy. Action commands are no more than *proprioceptive* predictions. Moreover, actions can be understood as being mediated by exactly the same mechanisms as exteroceptive predictions or perceptions (Adams et al., 2013). Histological data support this hypothesis. Specifically, the infragranular layers in the motor cortex and primary sensory neurons (projecting from muscle spindles to the dorsal horn of the spinal cord) comprise prediction units whereas alpha-motor neurons represent proprioceptive PEs units. Both types of proprioceptive predictions are compared in the ventral horn of the spinal cord, resulting in a proprioceptive PE that is minimized via discharges of alpha-motor neurons. Notice that whereas exteroceptive PEs minimization takes place in the granular layers of the cortex, proprioceptive PEs mimization takes place in the ventral horn of the spinal cord via alpha-motor neurons discharges. This histological difference between both systems accounts for the agranular property of the primary motor area (Adams et al., 2013; Shipp et al., 2013).

It follows that in pursuing adaptive homeostatic responses, *optimal* free-energy minimization must comprise explaining away exteroceptive and proprioceptive PEs in coordination. Echopraxia exemplifies this homeostatic need. An organism experiences echopraxia when it simultaneously perceives (observes) and executes an action, meaning, in the context of predictive coding, that exteroceptive and proprioceptive PEs are being simultaneously minimized. To counteract echopraxia (because it is not an adaptive homeostatic response), one type of PEs should not be minimized while the other is being explained away. In the context of active inference, this is exemplified by the dual physiological role of the so-called mirror neurons (Shipp et al., 2013) in the motor cortex. Mirror neurons fire when a primate either executes an action or observes the execution of such action. However, they do not fire when the primate simultaneously executes and observes the action.

In the current paradigm, a reactive response elicited by a task-switching demand decreases proprioceptive precision (increases uncertainty). This is because large proprioceptive PEs result from the comparison between the highly precise ongoing or prepotent response and the descending predictions of the unprepared behavior. Mechanistically, the reactive response translates into descending predictions originating in the infragranular layer of the primary motor cortex that opposes primary somatosensory signals, resulting in large and imprecise PEs (i.e., uncertainty). We speculate that if this situation occurs when exteroceptive PEs are minimized (e.g., in the 0-ms temporal gap condition), the organism successfully minimizes the proprioceptive PEs, resulting in successful task switching. In contrast, if this situation occurs in the context of large and not minimized PEs (e.g., in the 300-ms temporal gap condition), the organism increases free energy (analog to what happens during episodes of echopraxia) which is not an adaptive homeostatic response. Therefore, a predictive-coding based mechanism explaining how temporal PEs affects task-switching performance accuracy should include the coordination between exteroceptive and proprioceptive PEs minimization.

### A Predictive Coding Mechanism to Account for the Conjoint Effect of Temporal Gaps and PEs on Behavior-Change Performance

It is possible that in the presence of a large temporal gap (e.g., long delay) the extereoceptive process would reach a suboptimal state (large PEs without minimization). Triggering an *anticipatorily* prepared action minimizes additional free energy and compensates for this suboptimal state. A neurophysiological mechanism implementing this compensatory (i.e., homeostatic) response must satisfy two conditions. First, it must integrate exteroceptive and proprioceptive minimizations of PEs, which is no more than the integration of perception and action in a simple mechanism. Second, it must include the effects of temporal gaps (i.e., temporal uncertainty) and temporal PEs as driving or modulatory inputs.

The first condition is fulfilled with the fact that once the temporal PEs depart from lower level sensory areas and reach higher level areas such as the rAI, they would affect motor regions. Not coincidentally, the rAI is involved in the processing of both temporal PEs and in the fronto-basal ganglia circuit of motor control (Bolam, 2010) which is critical for successful task switching. The fronto-basal ganglia circuit is engaged during behavior inhibition^1^ (Aron et al., 2007). When an organism engages a behavior inhibition, a GO process (i.e., the prepotent behavior) triggered by a GO signal competes against a STOP process triggered by a STOP signal. Each process has a finishing time. Inhibition would be successful if the STOP process reaches its finishing time before the GO process.

Successful inhibition is associated with the activity of either the indirect or the hyperdirect fronto-basal ganglia circuit (Aron et al., 2007). It is relevant that behavior inhibition is an essential stage of task switching (Verbruggen et al., 2008; Verbruggen and Logan, 2009). In a stop-change task (Verbruggen et al., 2008; Verbruggen and Logan, 2009), the organism stops the prepotent behavior (i.e., GO1 behavior) before preparing a second behavior (i.e., GO2 behavior). Therefore, our task-switching paradigm might activate the frontostriatal network. A salient feature in this mechanism is that the rAI shows strong activity when the participant fails to inhibit responses (Cai et al., 2014). Furthermore, the rAI has anatomical connections with the presupplementary motor area and with the striatum which are part of the indirect pathway mediating effective behavior inhibition. Therefore, we suggest that the rAI anatomically connects “exteroceptive-related” circuits with “proprioceptive-related” circuits in a single network (Figure 6).

The second condition is fulfilled by the fact that both temporal gaps and temporal PEs might affect the effective connectivity (i.e., how the regions affect to each other) of the fronto-basal ganglia circuit (Figure 6). Based on our current data and our previous work (Limongi et al., 2013), we predict that whereas temporal uncertainty (caused by temporal gaps) would drive activity in the rAI, the temporal PEs would modulate the effective connections of the fronto-basal ganglia circuit. If proven true at the neurophysiological level, this mechanism would account for the modulatory effect of temporal PEs on task-switching performance that we found in this work.

### Conclusion

In his Principles of Psychology, James (1950) proposed that action follows perception which in modern neuroscience is referred to as the perception and action cycle. As a corollary, an accurate action demands an accurate perception. In consequence, in the temporal domain, the organism privileges temporal perception before engaging an action. Inaccurate temporal perceptions (i.e., temporal predictions) translate into large and not minimized PEs. These errors must be minimized before engaging an action. Therefore, the system must privilege the exteroceptive error minimization over other tasks (i.e., engaging a new and “uprepared” behavior). From a free energy perspective, we could interpret the irreversible (inaccurate) prepotent behavior as a compensation for imprecise perceptual inference. In other words, the brain calls upon precise prior beliefs (prepotent responses) when faced with imprecise sensory information so that to minimize the left-over free energy associated with the suboptimal Bayes estimate of the sensorium’s causes (i.e., not minimized exteroceptive PEs).

## Author Contributions

RL designed the study, performed the data analysis, and wrote the manuscript. AS designed the study, performed pilot data collection, and critically reviewed the manuscript. BG performed final data collection and critically reviewed the manuscript.

### Conflict of Interest Statement

The authors declare that the research was conducted in the absence of any commercial or financial relationships that could be construed as a potential conflict of interest.

## Footnotes

1 The action control literature uses the term “response inhibition.”

## References

[B1] Adams R. A. Perrinet L. U. Friston K. J. (2012). Smooth pursuit and visual occlusion: active inference and oculomotor control in schizophrenia. PLoS ONE 7:e47502. 10.1371/journal.pone.0047502 23110076 PMC3482214

[B2] Adams R. A. Shipp S. Friston K. J. (2013). Predictions not commands: active inference in the motor system. Brain Struc. Funct. 218, 611–643. 10.1007/s00429-012-0475-5 23129312 PMC3637647

[B3] Alexander W. H. Brown J. W. (2010). Computational models of performance monitoring and cognitive control. Top. Cogn. Sci. 2, 658–677. 10.1111/j.1756-8765.2010.01085.x 21359126 PMC3044326

[B4] Alexander W. H. Brown J. W. (2011). Medial prefrontal cortex as an action-outcome predictor. Nat. Neurosci. 14, 1338–1344. 10.1038/nn.2921 21926982 PMC3183374

[B5] Anderson D. R. (2008). Model Based Inference in Life Sciences: A Primer on Evidence. New York: Springer.

[B6] Aron A. R. Durston S. Eagle D. M. Logan G. D. Stinear C. M. Stuphorn V. (2007). Converging evidence for a fronto-basal-ganglia network for inhibitory control of action and cognition. J. Neurosci. 27, 11860–11864. 10.1523/JNEUROSCI.3644-07.2007 17978025 PMC6673355

[B7] Aron A. R. Poldrack R. A. (2006). Cortical and subcortical contributions to stop signal response inhibition: role of the subthalamic nucleus. J. Neurosci. 26, 2424–2433. 10.1523/JNEUROSCI.4682-05.2006 16510720 PMC6793670

[B8] Aron A. R. Robbins T. W. Poldrack R. A. (2014). Inhibition and the right inferior frontal cortex: one decade on. Trends Cogn. Sci. 18, 177–185. 10.1016/j.tics.2013.12.003 24440116

[B9] Badler J. B. Lefèvre P. Missal M. (2010). Causality attribution biases oculomotor responses. J. Neurosci. 30, 10517–10525. 10.1523/JNEUROSCI.1733-10.2010 20685994 PMC6634668

[B10] Badler J. B. Lefèvre P. Missal M. (2012). Divergence between oculomotor and perceptual causality. J. Vis. 12, 3. 10.1167/12.5.3 22593089

[B11] Balota D. A. Yap M. J. (2011). Moving beyond the mean in studies of mental chronometry: the power of response time distributional analyses. Curr. Dir. Psychol. Sci. 20, 160–166. 10.1177/0963721411408885

[B12] Bastos-Leite A. J. Ridgway G. R. Silveira C. Norton A. Reis S. Friston K. J. (2015). Dysconnectivity within the default mode in first-episode schizophrenia: a stochastic dynamic causal modeling study with functional magnetic resonance imaging. Schizophr. Bull. 41, 144–153. 10.1093/schbul/sbu080 24939881 PMC4266292

[B13] Bolam J. P. (2010). “Microcircuits of the striatum,” in Handbook of Brain Microcircuits, eds Shepherd G. M. Grillner S. (New York: Oxford University Press), 109–119.

[B14] Bossaerts P. (2010). Risk and risk prediction error signals in anterior insula. Brain Struc. Funct. 214, 645–653. 10.1007/s00429-010-0253-1 20512378

[B15] Botvinick M. Nystrom L. E. Fissell K. Carter C. S. Cohen J. D. (1999). Conflict monitoring versus selection-for-action in anterior cingulate cortex. Nature 402, 179–181. 10.1038/46035 10647008

[B16] Braver T. S. Barch D. M. Cohen J. D. (1999). Cognition and control in schizophrenia: a computational model of dopamine and prefrontal function. Biol. Psychiatry 46, 312–328. 10.1016/S0006-3223(99)00116-X 10435197

[B17] Brown J. W. (2008). Multiple cognitive control effects of error likelihood and conflict. Psychol. Res. 73, 744–750. 10.1007/s00426-008-0198-7 19030873 PMC3004025

[B18] Brown J. W. (2011). Medial prefrontal cortex activity correlates with time-on-task: what does this tell us about theories of cognitive control? Neuroimage 57, 314–315. 10.1016/j.neuroimage.2011.04.028 21540116

[B19] Brown J. W. Braver T. S. (2005). Learned predictions of error likelihood in the anterior cingulate cortex. Science 307, 1118–1121. 10.1126/science.1105783 15718473

[B20] Bryden D. W. Johnson E. E. Tobia S. C. Kashtelyan V. Roesch M. R. (2011). Attention for learning signals in anterior cingulate cortex. J. Neurosci. 31, 18266–18274. 10.1523/JNEUROSCI.4715-11.2011 22171031 PMC3285822

[B21] Cai W. Ryali S. Chen T. Li C.-S. R. Menon V. (2014). Dissociable roles of right inferior frontal cortex and anterior insula in inhibitory control: evidence from intrinsic and task-related functional parcellation, connectivity, and response profile analyses across multiple datasets. J. Neurosci. 34, 14652–14667. 10.1523/JNEUROSCI.3048-14.2014 25355218 PMC4212065

[B22] Carter C. S. Braver T. S. Barch D. M. Botvinick M. M. Noll D. Cohen J. D. (1998). Anterior cingulate cortex, error detection, and the online monitoring of performance. Science 280, 747–749. 10.1126/science.280.5364.747 9563953

[B23] Cohen J. D. Braver T. S. Brown J. W. (2002). Computational perspectives on dopamine function in prefrontal cortex. Curr. Opin. Neurobiol. 12, 223–229. 10.1016/S0959-4388(02)00314-8 12015241

[B24] Daunizeau J. Lemieux L. Vaudano A. E. Friston K. J. Stephan K. E. (2012). An electrophysiological validation of stochastic DCM for fMRI. Front. Comput. Neurosci. 6:103. 10.3389/fncom.2012.00103 23346055 PMC3548242

[B25] Eckert M. A. Menon V. Walczak A. Ahlstrom J. Denslow S. Horwitz A. (2009). At the heart of the ventral attention system: the right anterior insula. Hum. Brain Mapp. 30, 2530–2541. 10.1002/hbm.20688 19072895 PMC2712290

[B26] Fischer R. Plessow F. Ruge H. (2012). Priming of visual cortex by temporal attention? The effects of temporal predictability on stimulus(-specific) processing in early visual cortical areas. Neuroimage 66c, 261–269. 10.1016/j.neuroimage.2012.10.091 23142070

[B27] Friston K. J. (2009). The free-energy principle: a rough guide to the brain? Trends. Cogn. Sci. 13, 293–301. 10.1016/j.tics.2009.04.005 19559644

[B28] Friston K. J. (2010). The free-energy principle: a unified brain theory? Nat. Rev. Neurosci. 11, 127–138. 10.1038/nrn2787 20068583

[B29] Friston K. J. Daunizeau J. Kilner J. Kiebel S. J. (2010). Action and behavior: a free-energy formulation. Biol. Cybern. 102, 227–260. 10.1007/s00422-010-0364-z 20148260

[B30] Friston K. J. Kiebel S. J. (2009). Predictive coding under the free-energy principle. Philos. Trans. R. Soc. Lond. B Biol. Sci. 364, 1211–1221. 10.1098/rstb.2008.0300 19528002 PMC2666703

[B31] Friston K. J. Kilner J. Harrison L. (2006). A free energy principle for the brain. J. Physiol. Paris 100, 70–87. 10.1016/j.jphysparis.2006.10.001 17097864

[B32] Friston K. J. Stephan K. E. (2007). Free-energy and the brain. Synthese 159, 417–458. 10.1007/s11229-007-9237-y 19325932 PMC2660582

[B33] Gershman S. J. Blei D. M. Niv Y. (2010). Context, learning, and extinction. Psychol. Rev. 117, 197–209. 10.1037/a0017808 20063968

[B34] Hazy T. E. Frank M. J. O’Reilly R. C. (2006). Banishing the homunculus: making working memory work. Neuroscience 139, 105–118. 10.1016/j.neuroscience.2005.04.067 16343792

[B35] Hazy T. E. Frank M. J. O’Reilly R. C. (2007). Towards an executive without a homunculus: computational models of the prefrontal cortex/basal ganglia system. Philos. Trans. R. Soc. B Biol. Sci. 362, 1601–1613. 10.1098/rstb.2007.2055 17428778 PMC2440774

[B36] Jahfari S. Verbruggen F. Frank M. J. Waldorp L. J. Colzato L. Ridderinkhof K. R. (2012). How Preparation Changes the Need for Top-Down Control of the Basal Ganglia When Inhibiting Premature Actions. J. Neurosci. 32, 10870–10878. 10.1523/jneurosci.0902-12.2012 22875921 PMC6621019

[B37] James W. (1950). The Principles of Psychology. New York: Dover Publications.

[B38] Jones C. L. Minati L. Harrison N. A. Ward J. Critchley H. D. (2011). Under pressure: response urgency modulates striatal and insula activity during decision-making under risk. PLoS ONE 6:e20942. 10.1371/journal.pone.0020942 21677769 PMC3108983

[B39] Jones C. L. Ward J. Critchley H. D. (2010). The neuropsychological impact of insular cortex lesions. J. Neurol. Neurosurg. Psychiatry 81, 611–618. 10.1136/jnnp.2009.193672 20522870

[B40] Kahan J. Foltynie T. (2013). Understanding DCM: ten simple rules for the clinician. Neuroimage 83C, 542–549. 10.1016/j.neuroimage.2013.07.008 23850463

[B41] Kruschke J. (2008). Bayesian approaches to associative learning: from passive to active learning. Learn. Behav. 36, 210–226. 10.3758/LB.36.3.210 18683466

[B42] Limongi R. Sutherland S. C. Zhu J. Young M. E. Habib R. (2013). Temporal prediction errors modulate cingulate-insular coupling. Neuroimage 71, 147–157. 10.1016/j.neuroimage.2012.12.078 23333417

[B43] Limongi R. Tomio A. Ibanez A. (2014). Dynamical predictions of insular hubs for social cognition and their application to stroke. Front. Behav. Neurosci. 8:380. 10.3389/fnbeh.2014.00380 25408640 PMC4219475

[B44] Los S. A. (2013). The role of response inhibition in temporal preparation: evidence from a go/no-go task. Cognition 129, 328–344. 10.1016/j.cognition.2013.07.013 23969298

[B45] Menon V. Uddin L. Q. (2010). Saliency, switching, attention and control: a network model of insula function. Brain Struct. Funct. 214, 655–667. 10.1007/s00429-010-0262-0 20512370 PMC2899886

[B46] Myung J. I. (2000). The importance of complexity in model selection. J. Math. Psychol. 44, 190–204. 10.1006/jmps.1999.1283 10733864

[B47] Myung J. I. Pitt M. A. (2004). “Model comparison methods,” in Methods in Enzymology, eds Ludwig B. Michael L. J. (San Diego, CA: Academic Press), 351–366. 10.1016/S0076-6879(04)83014-3 15063657

[B48] Myung J. I. Tang Y. Pitt M. A. (2009). “Evaluation and comparison of computational models,” in Methods in Enzymology, eds Michael L. J. Ludwig B. (San Diego, CA: Academic Press), 287–304. 10.1016/S0076-6879(08)03811-1 PMC2704205 19216931

[B49] Nelson S. M. Dosenbach N. U. Cohen A. L. Wheeler M. E. Schlaggar B. L. Petersen S. E. (2010). Role of the anterior insula in task-level control and focal attention. Brain Struct. Funct. 214, 669–680. 10.1007/s00429-010-0260-2 20512372 PMC2886908

[B50] Nobre A. C. Correa A. Coull J. T. (2007). The hazards of time. Curr. Opin. Neurobiol. 17, 465–470. 10.1016/j.conb.2007.07.006 17709239

[B51] Nursimulu A. D. Bossaerts P. (2014). Risk and reward preferences under time pressure. Rev. Financ. 18, 999–1022. 10.1093/rof/rft013

[B52] O’Reilly R. C. Frank M. J. (2006). Making working memory work: a computational model of learning in the prefrontal cortex and basal ganglia. Neural Comput. 18, 283–328. 10.1162/089976606775093909 16378516

[B53] O’Reilly R. C. Braver T. S. Cohen J. D. (1999). “A biologically based computational model of working memory,” in Models of Working Memory: Mechanisms of Active Maintenance and Executive Control, eds Miyake A. Shah P. (Cambridge: Cambridge University Press), 375–411.

[B54] Payzan-LeNestour E. Bossaerts P. (2011). Risk, unexpected uncertainty, and estimation uncertainty: Bayesian learning in unstable settings. PLoS Comput. Biol. 7:e1001048. 10.1371/journal.pcbi.1001048 21283774 PMC3024253

[B55] Payzan-LeNestour E. Bossaerts P. (2012). Do not bet on the unknown versus try to find out more: estimation uncertainty and “unexpected uncertainty” both modulate exploration. Front. Neurosci. 6:150. 10.3389/fnins.2012.00150 23087606 PMC3472893

[B56] Payzan-LeNestour E. Dunne S. Bossaerts P. O’Doherty J. P. (2013). The neural representation of unexpected uncertainty during value-based decision making. Neuron 79, 191–201. 10.1016/j.neuron.2013.04.037 23849203 PMC4885745

[B57] Pitt M. A. Myung J. I. Zhang S. (2002). Toward a method of selecting among computational models of cognition. Psychol. Rev. 109, 472–491. 10.1037/0033-295X.109.3.472 12088241

[B58] Poldrack R. A. (2006). Can cognitive processes be inferred from neuroimaging data? Trends Cogn. Sci. 10, 59–63. 10.1016/j.tics.2005.12.004 16406760

[B59] Poldrack R. A. (2011). Inferring mental states from neuroimaging data: from reverse inference to large-scale decoding. Neuron 72, 692–697. 10.1016/j.neuron.2011.11.001 22153367 PMC3240863

[B60] Preuschoff K. Quartz S. R. Bossaerts P. (2008). Human insula activation reflects risk prediction errors as well as risk. J. Neurosci. 28, 2745–2752. 10.1523/JNEUROSCI.4286-07.2008 18337404 PMC6670675

[B61] Rescorla R. A. Wagner A. R. (1972). “A theory of Pavlovian conditioning: variations in the effectiveness of reinforcement and nonreinforcement,” in Classical Conditioning II: Current Research and Theory, eds Black A. H. Prokasy W. F. (New York: Appleton-Century-Crofts), 64–99.

[B62] Rohenkohl G. Gould I. C. Pessoa J. Nobre A. C. (2014). Combining spatial and temporal expectations to improve visual perception. J. Vis. 14, 1–13. 10.1167/14.4.8 24722562 PMC3983934

[B63] Sarinopoulos I. Grupe D. W. Mackiewicz K. L. Herrington J. D. Lor M. Steege E. E. (2010). Uncertainty during anticipation modulates neural responses to aversion in human insula and amygdala. Cereb. Cortex 20, 929–940. 10.1093/cercor/bhp155 19679543 PMC2837092

[B64] Schultz W. Preuschoff K. Camerer C. Hsu M. Fiorillo C. D. Tobler P. N. (2008). Explicit neural signals reflecting reward uncertainty. Philos. Trans. R. Soc. Lond. B Biol. Sci. 363, 3801–3811. 10.1098/rstb.2008.0152 18829433 PMC2581779

[B65] Shipp S. Adams R. A. Friston K. J. (2013). Reflections on agranular architecture: predictive coding in the motor cortex. Trends Neurosci. 36, 706–716. 10.1016/j.tins.2013.09.004 24157198 PMC3858810

[B66] Symmonds M. Moran R. J. Wright N. D. Bossaerts P. Barnes G. Dolan R. J. (2013). The chronometry of risk processing in the human cortex. Front. Neurosci. 7:146. 10.3389/fnins.2013.00146 23970849 PMC3747673

[B67] Verbruggen F. Logan G. D. (2009). Models of response inhibition in the stop-signal and stop-change paradigms. Neurosci. Biobehav. Rev. 33, 647–661. 10.1016/j.neubiorev.2008.08.014 18822313 PMC2696813

[B68] Verbruggen F. Schneider D. W. Logan G. D. (2008). How to stop and change a response: the role of goal activation in multitasking. J. Exp. Psychol. Hum. Percept. Perform. 34, 1212–1228. 10.1037/0096-1523.34.5.1212 18823206

[B69] Wagenmakers E.-J. Farrell S. (2004). AIC model selection using Akaike weights. Psychon. Bull. Rev. 11, 192–196. 10.3758/BF03206482 15117008

[B70] Young M. E. Rogers E. T. Beckmann J. S. (2005). Causal impressions: predicting when, not just whether. Mem. Cogn. 33, 320–331. 10.3758/BF03195320 16028586

[B71] Young M. E. Sutherland S. (2009). The spatiotemporal distinctiveness of direct causation. Psychon. Bull. Rev. 16, 729–735. 10.3758/PBR.16.4.729 19648460

